# Community-associated Methicillin-Resistant *Staphylococcus aureus* Strains in Pediatric Intensive Care Unit^1^

**DOI:** 10.3201/eid1604.090107

**Published:** 2010-04

**Authors:** Aaron M. Milstone, Karen C. Carroll, Tracy Ross, K. Alexander Shangraw, Trish M. Perl

**Affiliations:** The Johns Hopkins University School of Medicine, Baltimore, Maryland, USA

**Keywords:** methicillin resistance, antimicrobial resistance, *Staphylococcus aureus*, pediatric intensive care units, transmission, epidemiology, prevalence, bacteria, research

## Abstract

Endemicity may lead to increased resistance and virulence.

## CME ACTIVITY

Medscape, LLC is pleased to provide online continuing medical education (CME) for this journal article, allowing clinicians the opportunity to earn CME credit. This activity has been planned and implemented in accordance with the Essential Areas and policies of the Accreditation Council for Continuing Medical Education through the joint sponsorship of Medscape, LLC and Emerging Infectious Diseases. Medscape, LLC is accredited by the ACCME to provide continuing medical education for physicians. Medscape, LLC designates this educational activity for a maximum of 0.5 *AMA PRA Category 1 Credits*™. Physicians should only claim credit commensurate with the extent of their participation in the activity. All other clinicians completing this activity will be issued a certificate of participation. To participate in this journal CME activity: (1) review the learning objectives and author disclosures; (2) study the education content; (3) take the post-test and/or complete the evaluation at www.medscapecme.com/journal/eid; (4) view/print certificate.

## Learning Objectives

Upon completion of this activity, participants will be able to:

- Identify risk factors among children for being a community-acquired methicillin-resistant *Staphylococcus aureus* (CA-MRSA) carrier.
- Recognize the benefits of screening for MRSA colonization in children being admitted to the hospital.
- Predict a consequence of undetected CA-MRSA carriers admitted to a hospital setting.

## Editor

**Carol Snarey,** Copyeditor, *Emerging Infectious Diseases. Disclosure: Carol Snarey has disclosed no relevant financial relationships.*

## CME AUTHOR

**Charles P. Vega, MD**, Associate Professor; Residency Director, Department of Family Medicine, University of California, Irvine, CA, USA. *Disclosure: Charles P. Vega, MD, has disclosed no relevant financial relationships.*

## AUTHORS

Disclosures**: Aaron M. Milstone, MD, MHS**, has disclosed the following relevant financial relationship: received grants for clinical research from Sage Products, Inc. **Karen C. Carroll, MD**, has disclosed the following relevant relationships: served as an advisor or consultant for Quidel Diagnostics; OpGen, Inc.; Boehringer Ingelheim Pharmaceuticals, Inc.; received grants for clinical research from BD GeneOhm; Ibis Biosciences, Inc.; MicroPhage, Inc. **Tracy Ross, BS,** and **K. Alexander Shangraw, MSPH,** have disclosed no relevant financial relationships. **Trish M. Perl, MD, MSc**, has disclosed the following relevant financial relationships: served as an advisor or consultant for Cadence Pharmaceuticals; 3M; TheraDoc Inc.; received grants for clinical research from US Centers for Disease Control and Prevention; Merck & Co., Inc.; Sage Products, Inc.; US Department of Veterans Affairs.
