# Supplementary material for: Community-associated Methicillin-Resistant Staphylococcus aureus Strains in Pediatric Intensive Care Unit
Source: Emerg Infect Dis. 2010 Apr;16(4):647–55. doi: 10.3201/eid1604.090107 (PMC3321932; doi:10.3201/eid1604.090107)
Supplement: Related texts

# Research article: Community-associated Methicillin-Resistant *Staphylococcus aureus* Strains in Pediatric Intensive Care Unit^1^

**DOI:** 10.3201/eid1604.090107

**Authors:** Aaron M. Milstone, Karen C. Carroll, Tracy Ross, K. Alexander Shangraw, Trish M. Perl

**Affiliations:** The Johns Hopkins University School of Medicine, Baltimore, Maryland, USA

## Abstract

Virulent community-associated methicillin-resistant *Staphylococcus-aureus* (CA-MRSA) strains have spread rapidly in the United States. To characterize the degree to which CA-MRSA strains are imported into and transmitted in pediatric intensive care units (PICU), we performed a retrospective study of children admitted to The Johns Hopkins Hospital PICU, March 1, 2007–May 31, 2008. We found that 72 (6%) of 1,674 PICU patients were colonized with MRSA. MRSA-colonized patients were more likely to be younger (median age 3 years vs. 5 years; p = 0.02) and African American (p<0.001) and to have been hospitalized within 12 months (p<0.001) than were noncolonized patients. MRSA isolates from 66 (92%) colonized patients were fingerprinted; 40 (61%) were genotypically CA-MRSA strains. CA-MRSA strains were isolated from 50% of patients who became colonized with MRSA and caused the only hospital-acquired MRSA catheter-associated bloodstream infection in the cohort. Epidemic CA-MRSA strains are becoming endemic to PICUs, can be transmitted to hospitalized children, and can cause invasive hospital-acquired infections. Further appraisal of MRSA control is needed.

## Community-associated Methicillin-Resistant *Staphylococcus aureus* Strains in Pediatric Intensive Care Unit^1^

Methicillin-resistant *Staphylococcus aureus* (MRSA) frequently infects children. Traditionally, MRSA infections were confined to those who frequented healthcare facilities or had predisposing healthcare-associated risk factors. In the 1990s, reports emerged of MRSA infections in healthy children in the community who had no predisposing risk factors (*1*). Community-onset MRSA infections were caused by MRSA strains belonging to the genotypes USA300 and USA400 (identified by pulsed-field gel electrophoresis [PFGE]), also referred to as the community-associated MRSA (CA-MRSA) strains (*2*,*3*). These CA-MRSA strains are associated with increased production of toxins and are less resistant to antimicrobial drugs than are traditional hospital-acquired MRSA (HA-MRSA) strains (*4*,*5*). Although CA-MRSA strains usually cause mild skin and soft tissue infections, they can also cause severe and fatal disease (*6*–*8*).

As the community prevalence of MRSA has risen (*9*), more children colonized or infected with MRSA have been admitted to hospitals (*10*–*12*), especially with phenotypic CA-MRSA strains. Notably, CA-MRSA strains can cause outbreaks in hospitals (*13*) and have become a frequent cause of hospital-onset infections (*14*,*15*). Aside from ways to manage outbreaks (*16*) and a report that clinical cultures underestimate MRSA prevalence (*17*), little is known about the prevalence of MRSA colonization of hospitalized children. The degree to which CA-MRSA strains are imported into and transmitted in high-risk settings such as pediatric intensive care units (PICUs) has not been determined. Understanding the effects of MRSA in hospitalized children is essential to guide, assess, and plan MRSA prevention and control programs among hospitalized children. Our objectives were to measure the prevalence of MRSA colonization at the time of admission to the PICU and to determine the effects of CA-MRSA strains on MRSA colonization, transmission, and hospital-acquired infections in the PICU.

## Materials and Methods

### Setting and Design

The Johns Hopkins Hospital is a 920-bed tertiary care academic medical center with an embedded 175-bed children’s hospital. The institution serves Baltimore, Maryland, USA, and the surrounding area. The 26-bed PICU admits ≈1,700 medical and surgical patients each year, including patients needing hematopoietic stem cell transplants and organ transplants, as well as cardiac, orthopedic, and neurosurgical patients. Beginning March 1, 2007, as part of a hospital MRSA prevention and control program, the Department of Hospital Epidemiology and Infection Control initiated screening of patients for MRSA colonization at the time of admission to the PICU and weekly thereafter. Nares swab specimens were obtained by PICU nurses and cultured for MRSA as described later. Newly identified patients or those known to be colonized or infected with MRSA were isolated in cohort groups. Compliance with admission screening was reported back to the unit monthly. Hospital policy required strict hand hygiene, use of standard precautions, and contact isolation for all patients colonized or infected with MRSA.

During March 1, 2007–May 31, 2008, we performed a retrospective cohort study to identify all MRSA-colonized patients in the Johns Hopkins Hospital PICU, including those colonized at the time of admission and those who became colonized while in the PICU. If patients were admitted to the PICU multiple times, only the first admission was included. The institutional review board approved this study and waived informed consent to review retrospective data collected during hospital care.

### Definitions

MRSA colonization at the time of PICU admission was defined as having a nasal surveillance culture obtained at the time of admission that grew MRSA or any clinical culture that grew MRSA within 3 days of PICU admission (*18*,*19*). Known MRSA carriers were defined as any patients with an institutional history of MRSA colonization or infection before PICU admission. Newly identified MRSA patients (incident cases) had no institutional history of MRSA colonization or infection (either had negative cultures on prior admissions or clinic visits or had not previously been tested). Case-patients with incident MRSA became colonized or infected in the PICU and met the following criteria: 1) had a positive screening or clinical culture obtained >3 days after admission to the PICU (*19*), 2) had no institutional history of MRSA, and 3) had a previous negative culture from the same site during the current PICU admission. Incidence density was calculated as the number of incident MRSA cases per 1,000 patient-days at risk for MRSA acquisition (i.e., patient days during which patients were not known to be colonized or infected with MRSA). Healthcare-associated MRSA (HA-MRSA) infections met the criteria established by the National Healthcare Safety Network’s surveillance definition for healthcare-associated infections (*20*). CA-MRSA strains included those belonging to the PFGE genotypes USA300 and USA400 (*3*). HA-MRSA strains included those belonging to other PFGE genotypes.

### Data Collection and Case Identification

We searched a computerized surveillance support system (Theradoc Inc., Salt Lake City, UT, USA) to identify all patients with microbiology cultures of samples from any body site that grew MRSA from March 1, 2007, through May 31, 2008, and to determine compliance in performing screening cultures at the time of PICU admission. Administrative databases were searched to obtain patient characteristics. Medical records were reviewed to determine whether MRSA infections met the National Healthcare Safety Network’s surveillance definition for healthcare-associated infections.

### Laboratory Methods

Nasal surveillance swabs were plated on BBL CHROMagar MRSA plates (BD Diagnostics, Sparks, MD, USA), a selective and differential medium to detect MRSA. Mauve-colored colonies present after 24 or 48 hours of incubation were confirmed as *S. aureus* by Gram stain and slide coagulase testing (*21*). We performed PFGE on available stored isolates. DNA was extracted and digested by using *Sma*1 (*22*,*23*). We used *S. aureus* subspecies NCTC 8325 as a control strain, and all USA PFGE strain types were included for comparison (*3*). The USA type strains were obtained through the Network of Antimicrobial Resistance in *Staphylococcus aureus* program (supported under National Institute of Allergy and Infectious Diseases/National Institutes of Health contract no. HHSN272200700055C). We performed PFGE on the CHEF-DR III (BioRad Laboratories, Hercules, CA, USA). Gels were stained and scanned by using a molecular analysis fingerprinting software (Fingerprinting II Version 3.0; BioRad Laboratories). We considered isolates to be related if their patterns had <3 band differences (*3*) and to be unrelated if they had >3 band differences.

### Statistical Analysis

Data were maintained in Microsoft Access 2003 (Microsoft Corp., Redmond, WA, USA) and analyzed by using Stata version 10.0 (StataCorp., College Station, TX, USA). Means, medians, and interquartile ranges (IQRs) were calculated for select demographic variables. Categorical variables were expressed as numbers and percentages. Comparisons were made by using the Pearson χ^2^ or Fisher exact test. For continuous variables, the Student *t* test or the Wilcoxon rank-sum test was used to compare groups, depending on the distribution of the data. Logistic regression was used to calculate odds ratios (ORs) and 95% confidence intervals (CIs) and to evaluate the strength of associations. A pairwise correlation coefficient was calculated to assess an association between incident cases and monthly admission colonization prevalence. A 2-tailed p value <0.05 was considered significant for all statistical tests.

## Results

From March 1, 2007, through May 31, 2008, 1,674 children were admitted to the Johns Hopkins Hospital PICU. The median age was 5 years (IQR 1–12 years), and 55% of patients were male. Only 53 patients (3.2%) had an institutional history of MRSA colonization or infection. Screening cultures were performed on nasal swabs from 1,210 children (72%) obtained at the time of PICU admission. When patients that were screened were compared with those that were not screened, screened patients were more likely to have been hospitalized in the previous 12 months (29% vs. 22%, p<0.01). No other significant differences in demographic or clinical characteristics were found between those patients screened for MRSA colonization and those not screened (Table 1).

**Table 1:** Characteristics of patients screened for and not screened for MRSA colonization at the time of PICU admission, The Johns Hopkins University Hospital, Baltimore, MD, USA, March 2007–May 2008*

Characteristic	Patients screened for MRSA, n = 1,210	Patients not screened for MRSA, n = 464	p value
Demographic			
Median age, y (IQR)	5 (0–12)	6 (1–12)	0.28
Male sex	667 (55)	255 (56)	0.78
Race			
White	676 (56)	234 (51)	Referent†
African American	403 (33)	173 (37)	0.07
Other	131 (11)	57 (12)	0.19
Clinical			
Known MRSA carrier‡	41 (3)	12 (3)	0.40
Hospitalized in previous 12 mo	355 (29)	102 (22)	<0.01

*MRSA, methicillin-resistant *Staphylococcus aureus*; PICU, pediatric intensive care unit; IQR, interquartile range. Values reported as no. (%) unless otherwise specified. †Obtained from univariate logistic regression analysis. ‡Patients with institutional history of MRSA colonization or infection.

At the time of admission to PICU, 72 (6.0%) patients were colonized with MRSA: 68 patients (94%) identified by results of nasal screening cultures and 4 patients whose clinical culture grew MRSA within 3 days of PICU admission. Characteristics of patients colonized with MRSA at the time of admission (group 1, currently colonized) were compared with those of patients not colonized with MRSA and never known to be colonized (group 2, never colonized) and patients not colonized with MRSA at admission but who had an institutional history of prior colonization or infection (group 3, previously colonized) (Table 2). Compared with never-colonized patients, MRSA-colonized patients tended to be younger (median age 3 years vs. 5 years, p = 0.02), to be African American (54% vs. 32%; p<0.001), and to have been hospitalized in the previous 12 months (p<0.001). MRSA-colonized patients had a longer stay in the PICU (3 days vs. 2 days, p<0.001) and a longer stay in the hospital (8 days vs. 5 days; p<0.01). MRSA-colonized patients tended to be younger than previously colonized patients (median age 3 years vs. 10.5 years; p<0.01), but these 2 groups were otherwise similar.

**Table 2:** Characteristics of patients with and without MRSA colonization at the time of PICU admission, The Johns Hopkins University Hospital, Baltimore, MD, USA, March 2007–May 2008*

Characteristic	Group 1,† n = 72	Group 2,‡ n = 1,117	Group 3, n = 24§	p value	
				Group 2 vs. group 1	Group 3 vs. group 1
Demographic					
Median age, y (IQR)	3 (0–7.5)	5 (1–12)	10.5 (3–13)	0.02	<0.01
Male sex	38 (53)	616 (55)	13 (54)	0.69	0.91
Race					
White	28 (39)	640 (57)	10 (42)	Referent¶	Referent¶
African American	39 (54)	352 (32)	13 (54)	<0.001	0.89
Other	5 (7)	126 (11)	1 (4)	0.86	0.62
Clinical					
Known MRSA carrier#	18 (25)	0 (0)	24 (100)		
Hospitalization in previous 12 mo	42 (58)	308 (28)	14 (58)	<0.001	0.41
Outcomes					
PICU length of stay,** median (IQR)	3 (1–7)	2 (1–4)	2.5 (1–9)	<0.001	0.96
Hospital length of stay,** median (IQR)	8 (3.5–15.5)	5 (3–10)	6 (3/5–14.5)	<0.01	0.70

*MRSA, methicillin-resistant Staphylococcus aureus; PICU, pediatric intensive care unit; IQR, interquartile range. Values reported as no. (%) unless otherwise specified. †MRSA colonized: patients who had MRSA grow in an admission nasal surveillance culture or in any clinical culture within 3 days of PICU admission. ‡Not MRSA colonized/no institutional history of MRSA colonization. §Not MRSA colonized/institutional history of MRSA colonization. ¶Obtained from univariate logistic regression analysis. #Patients with institutional history of MRSA colonization or infection. **Data were log transformed before regression analysis to account for skewing.

Of the 72 MRSA colonized patients, 54 (75%) were newly identified MRSA carriers (51 by screening cultures and 3 by clinical cultures). Of the 51 patients newly identified by screening cultures, 8 (16%) had a subsequent clinical culture grow MRSA during their PICU stay. Therefore, 43 (60%) of 72 MRSA-colonized patients would have gone undetected had admission screening cultures not been performed. Most MRSA-colonized patients were <6 years of age (71%) and African American (54%). Eighteen percent were admitted from an in-patient unit, and in the previous 12 months, 58% had been hospitalized and 43% had been admitted to the PICU.

MRSA isolates from 66 (92%) of 72 patients colonized with MRSA at the time of admission were analyzed by PFGE; 14 distinct strains were identified (Figure). Forty (61%) isolates were CA-MRSA strains, including those identical to or related to PFGE types USA300 (n = 39) and USA400 (n = 1). Twenty-six isolates (39%) were HA-MRSA strains related to USA100 (n = 11), USA200 (n = 1), USA700 (n = 1), and 8 other strains had unique PFGE fingerprints (n = 13). Many isolates did not have identical band patterns, but they did have <3 band differences and were considered related strains (*3*).

**Figure:** Dendrogram of methicillin-resistant *Staphylococcus aureus* strains that colonized children admitted to the pediatric intensive care unit, The Johns Hopkins Hospital, Baltimore, MD, USA, 2007–2008. Isolates were characterized by pulsed-field gel electrophoresis (PFGE). Not all strains within a PFGE type had identical patterns, but strains were considered related with <3 band differences; 66 isolates were analyzed. The number of isolates related to each PFGE type is listed. *Reference strains.

Patients colonized with CA-MRSA were compared with those colonized with HA-MRSA (Table 3). Patients colonized with CA-MRSA strains were less likely to have been admitted to the PICU within the previous 12 months (OR 0.31; 95% CI 0.11–0.84). No significant differences were found between the 2 groups in other demographic, clinical, or outcome characteristics, including median age, sex, number of newly identified MRSA carriers, length of stay in the hospital before PICU admission, admission from home or patient care unit, admission to a medical or surgical service, PICU length of stay, or hospital length of stay.

**Table 3:** Characteristics of patients colonized with different MRSA strain types at the time of PICU admission, The Johns Hopkins Hospital, Baltimore, MD, USA, March 2007–May 2008*

Characteristic	Patients colonized with CA-MRSA strain, n = 40	Patients colonized with HA-MRSA strain, n = 26	OR (95% CI)†
Demographic			
Median age, y (IQR)	3.8 (1.0–5.9)	4.0 (1.0–9.5)	0.98 (0.90–1.07)
Male sex	22 (55)	14 (54)	1.05 (0.39–2.82)
Race			
White	15 (35)	9 (38)	Referent
African American	23 (58)	13 (50)	1.1 (0.36–3.10)
Other	2 (5)	4 (15)	0.3 (0.05–1.98)
Clinical			
Newly identified MRSA carrier	32 (80)	20 (77)	1.2 (0.36–3.97)
Hospitalized in previous 12 mo	20 (50)	19 (73)	0.37 (0.13–1.07)
ICU admission in previous 12 mo	13 (33)	26 (62)	0.31 (0.11–0.84)
Length of stay in hospital before PICU admission, median (range)	0 (0–28)	0 (0–14)	1.02 (0.92–1.15)
Primary service			
Medical	24 (60)	12 (46)	Referent
Surgical	16 (40)	14 (54)	0.57 (0.21–1.55)
Admitted to PICU from inpatient unit	6 (19)	5 (15)	0.74 (0.20–2.73)
Outcomes			
PICU length of stay,‡ median (IQR)	3 (1–7.5)	3 (2–7)	1.05 (0.79–1.40)
Hospital length of stay,‡ median (IQR)	8 (4.5–28.5)	8.5 (3–15)	1.04 (0.97–2.04)

*MRSA, methicillin-resistant *Staphylococcus aureus*; PICU, pediatric intensive care unit; CA-MRSA, community-associated MRSA; HA-MRSA, hospital-associated MRSA; OR, odds ratio; CI, confidence interval; IQR, interquartile range. Values reported as no. (%) unless otherwise specified. †Obtained from univariate logistic regression analysis. ‡Data were log transformed before regression analysis to account for skewing.

To identify patients who became colonized with MRSA while in the PICU, MRSA screening cultures from nares specimens were sent weekly, and clinical cultures from patients in the PICU for >3 days were monitored. During the study period, 8 incident MRSA cases were identified (Table 4), an incidence density of 1.01 cases per 1,000 patient days at risk. No correlation was shown between monthly colonization prevalence at time of admission and incident MRSA cases (p = 0.09). Six (75%) of 8 incident cases were identified by a screening culture. Of these 6 patients, 2 had subsequent clinical cultures grow MRSA. If weekly MRSA screening cultures were not performed, only 4 (50%) of 8 incident MRSA cases would have been identified. Patients with incident MRSA cases were in the PICU for a median of 6 days (range 5–24 days) before acquiring MRSA and had a median age of 6 years (range 1–11 years). Seven (88%) of 8 MRSA isolates were available for PFGE analysis. Four (57%) of 7 isolates were identical to or related to PFGE-type USA300, documenting healthcare-associated transmission of CA-MRSA strains. Six (75%) of 8 patients with incident cases were admitted to a surgical service. Of the patients who became colonized with MRSA, 3 (38%) acquired a subsequent MRSA infection during their stay in the PICU (1 central-line–associated bloodstream infection, 1 case of ventilator-associated pneumonia, 1 case of ventilator-associated tracheitis). Both respiratory infections were caused by PFGE strain A, a strain that was not associated with colonization of any patients at the time of PICU admission. The only HA-MRSA bloodstream infection was caused by a USA300-related strain.

**Table 4:** Characteristics of patients who acquired MRSA colonization in the PICU, The Johns-Hopkins University Hospital, Baltimore, MD, USA, March 2007-2008.

Patient no.	Age, y	Culture type	Days in PICU before MRSA acquisition	Strain type	Clinical service
1	7.0	Clin	19	A	Surg
2	4.5	Surv	7	A	Med
3	11.2	Surv	5	B	Surg
4	2.5	Surv	9	USA300	Surg
5	1.2	Surv	5	USA300	Surg
6	9.9	Surv	24	USA300	Surg
7	3.8	Clin	5	USA300	Surg
8	7.5	Surv	5	Unknown	Med

*MRSA, methicillin-resistant *Staphylococcus aureus*; PICU, pediatric intensive care unit; Clin, clinical; Surg, surgical; Surv, surveillance; Med, medical.

## Discussion

These data describe the prevalence of MRSA colonization in patients admitted to the PICU and suggest that CA-MRSA strains may be becoming endemic in hospitalized children. We found that 6.0% of patients screened at the time of admission to the PICU were colonized with MRSA. Most (60%) MRSA-colonized patients would not have been recognized if admission screening cultures had not been performed. Sixty-one percent of MRSA colonized patients harbored CA-MRSA strains, mostly USA300. Our data show that epidemic CA-MRSA strains are endemic to the PICU. These strains can be transmitted to children in the hospital and can cause invasive hospital-acquired infections, including bacteremia.

Aside from how to manage an MRSA outbreak, little research has attempted to characterize the epidemiology of MRSA colonization and transmission in the PICU. The lack of research in PICU patients is surprising, given the abundance of published articles that have characterized the epidemiology of MRSA in adult ICU patients (*24*,*25*). Our findings agree with those of studies of adult patients: screening cultures detect a large reservoir of MRSA-colonized patients, cases that would otherwise go undetected (*18*,*24*). Most (75%) patients colonized at the time of PICU admission had no institutional history of MRSA colonization or infection. MRSA-colonized patients serve as a reservoir for contamination of healthcare workers’ hands and subsequent MRSA transmission to patients. Active detection and isolation of MRSA carriers can reduce MRSA transmission in hospitals (*26*,*27*). Given the risk to patients for MRSA acquisition and subsequent infection (*28*), many hospitals screen high-risk populations to identify MRSA carriers. In an attempt to curb the spread of MRSA in healthcare facilities, some states have passed legislation mandating MRSA screening.

As the community prevalence of MRSA has risen (*9*), more children infected with MRSA have been admitted to hospitals (*10*,*11*). Our data suggest that these patients represent a small fraction of the patients colonized with CA-MRSA strains who enter the hospital. We found that 61% of children colonized with MRSA at the time of PICU admission harbored a CA-MRSA strain. Findings from a previous cohort showed a lower percentage of colonization with CA-MRSA strains (*29*). Among patients >13 years of age who were admitted to an urban hospital in Atlanta, 7.3% were colonized with MRSA, and 30% of those were colonized with CA-MRSA strains (*29*).

Several factors may explain the high prevalence of colonization with CA-MRSA strains in our cohort. First, the community prevalence of MRSA colonization in children is increasing nationwide and in Baltimore (*30*–*32*), largely the result of spread of CA-MRSA strains. Second, children generally have less exposure to healthcare settings where they would be exposed to traditional HA-MRSA strains. Third, children frequently are in settings of close personal contact where opportunities for hygiene may be limited, such as day care, schools, and sports teams, settings postulated as high-risk environments for MRSA transmission.

Notably, children in this study who were colonized with MRSA at the time of admission to PICU were more likely to be younger and African American. Our finding of younger age is consistent with data from the 2003–2004 Centers for Disease Control and Prevention National Health and Nutrition Examination Survey Nasal Swab survey, which found that children 1–5 years of age had the second highest MRSA colonization rates, behind adults >60 years of age (R. Gorwitz, pers. comm.). Similarly, we found that children with previous, but not current, MRSA colonization tended to be older. Why colonization prevalence is different in various pediatric age groups remains unknown. Previous studies have found racial disparities in rates of invasive MRSA disease (*6*,*33*). In a 2007 report by Klevens et al., incidence rates of invasive MRSA disease were more than twice as high for African Americans than for whites, and mortality rates were 80% higher for African Americans (*7*). The reason for these racial disparities is unknown. MRSA colonization is a known risk factor for invasive MRSA, so higher colonization rates in African Americans may in part explain higher rates of invasive disease.

CA-MRSA strains are changing the landscape of MRSA infection prevention and control in hospitals. We found that CA-MRSA strain USA300 was the most commonly acquired MRSA strain identified in the PICU. All patients who acquired MRSA had negative nares swab cultures at the time of PICU admission, and all subsequently exhibited MRSA nasal colonization. Of 8 patients, 3 (38%) had subsequent MRSA infection during their PICU stay. We were unable to monitor these patients after PICU discharge and likely have underestimated their long-term risk for subsequent MRSA infection. CA-MRSA strains, with the potential to spread rapidly and cause severe disease, have now been shown to cause hospital-acquired infections and hospital outbreaks (*7*,*13*–*15*). Hospital outbreaks confirm that CA-MRSA strains can be transmitted and acquired in the healthcare setting. The role of CA-MRSA strains in endemic MRSA transmission has not been elucidated. The extent to which endemic CA-MRSA strains will affect the epidemiology of HA-MRSA transmission and infections remains unknown. Our data suggest that this topic requires further study.

As CA-MRSA strains become more prevalent in hospitals, the importance of distinguishing between MRSA strains remains unclear. However, CA-MRSA strains appear to be highly transmissible and may have increased virulence (*33*–*35*). When attempting to distinguish between patients colonized with CA-MRSA strains and those colonized with non–CA-MRSA strains, we found that patients colonized with HA-MRSA strains were 3× more likely to have been admitted to an ICU within the previous 12 months. Other demographic characteristics did not differ between the groups. Overall, our findings agree with those of other studies that have shown that demographic data and risk factors may not reliably distinguish between patients colonized or those infected with various MRSA strains (*14*,*36*).

Our study has several limitations. First, only nares cultures were performed to identify asymptomatic MRSA carriers at the time of admission to the PICU. Recent studies have shown that screening extranasal sites or subtances, such as throat, axilla, perineum, or stool can increase the detection of MRSA carriers (*37*,*38*), especially those colonized with CA-MRSA strains. However, the best sites to detect MRSA, in combination with nasal culture, remain unclear (*38*). Nares cultures, if poorly carried out, can have false-negative results. Therefore, if we misclassified MRSA carriers with negative nasal screening cultures, we may have underestimated the MRSA prevalence. Sensitive, yet cost-effective, methods of screening for MRSA colonization are still needed.

The second limitation is that admission screening cultures were instituted in March 2007, and compliance with screening was only 72%. Our PICU did not have admission order sets, and in the absence of a physician’s written order, cultures were not always performed. Compliance improved over time with initiation of a patient order entry system, a visual reminder to perform screening cultures on the front of the patient’s chart, and frequent compliance auditing by the nurse manager. However, given the similarities between screened and unscreened patients, we expect that our measured prevalence was representative of the entire cohort. Third, our PICU has low MRSA incidence rates and may have a low prevalence of MRSA colonization at the time of admission compared with other PICUs. These conditions may limit how our findings can be generalized to other institutions.

Overall, we found that epidemic CA-MRSA strains are likely endemic to PICUs. These virulent and transmissible strains are entering the PICU through infected or colonized patients, they are being transmitted to children, and they are responsible for hospital-onset MRSA infections. CA-MRSA strains often colonize children without healthcare-associated risk factors. Traditional infection-control strategies, in which risk factors are used to target high-risk patients for screening and intervention, may prove insufficient for MRSA. Future studies to determine optimal approaches to control MRSA transmission in hospitalized children are needed. As CA-MRSA strains enter the hospital environment, the increased frequency of methicillin resistance and the coexistence of multiple strain types may lead to the selection of novel MRSA strains with enhanced capacity for transmission and infection. These conditions would be especially concerning with regard to children, for whom a more restricted antimicrobial drug arsenal is available. Sound epidemiologic investigation and feasible interventions are needed to control MRSA and protect hospitalized children.

## Footnotes

1 These data were presented in part at the Annual Scientific Meeting of the Society of Healthcare Epidemiology of America, Orlando, Florida, USA, April 2008.

## References

[1] Herold BC, Immergluck LC, Maranan MC, Lauderdale DS, Gaskin RE, Boyle-Vavra S, Community-acquired methicillin-resistant *Staphylococcus aureus* in children with no identified predisposing risk. JAMA. 1998;279:593–8. 10.1001/jama.279.8.5939486753

[2] Daum RS, Ito T, Hiramatsu K, Hussain F, Mongkolrattanothai K, Jamklang M, A novel methicillin-resistance cassette in community-acquired methicillin-resistant *Staphylococcus aureus* isolates of diverse genetic backgrounds. J Infect Dis. 2002;186:1344–7. 10.1086/34432612402206

[3] McDougal LK, Steward CD, Killgore GE, Chaitram JM, McAllister SK, Tenover FC. Pulsed-field gel electrophoresis typing of oxacillin-resistant *Staphylococcus aureus* isolates from the United States: establishing a national database. J Clin Microbiol. 2003;41:5113–20. 10.1128/JCM.41.11.5113-5120.200314605147PMC262524

[4] Naimi TS, LeDell KH, Como-Sabetti K, Borchardt SM, Boxrud DJ, Etienne J, Comparison of community- and health care-associated methicillin-resistant *Staphylococcus aureus* infection. JAMA. 2003;290:2976–84. 10.1001/jama.290.22.297614665659

[5] Vandenesch F, Naimi T, Enright MC, Lina G, Nimmo GR, Heffernan H, Community-acquired methicillin-resistant *Staphylococcus aureus* carrying Panton-Valentine leukocidin genes: worldwide emergence. Emerg Infect Dis. 2003;9:978–84.1296749710.3201/eid0908.030089PMC3020611

[6] Fridkin SK, Hageman JC, Morrison M, Sanza LT, Como-Sabetti K, Jernigan JA, Methicillin-resistant *Staphylococcus aureus* disease in three communities. N Engl J Med. 2005;352:1436–44. 10.1056/NEJMoa04325215814879

[7] Klevens RM, Morrison MA, Nadle J, Petit S, Gershman K, Ray S, Invasive methicillin-resistant *Staphylococcus aureus* infections in the United States. JAMA. 2007;298:1763–71. 10.1001/jama.298.15.176317940231

[8] Agwu A, Brady KM, Ross T, Carroll KC, Halsey NA. Cholera-like diarrhea and shock associated with community-acquired methicillin-resistant *Staphylococcus aureus* (USA400 clone) pneumonia. Pediatr Infect Dis J. 2007;26:271–3. 10.1097/01.inf.0000256766.25247.d417484232

[9] Gorwitz RJ, Kruszon-Moran D, McAllister SK, McQuillan G, McDougal LK, Fosheim GE, Changes in the prevalence of nasal colonization with *Staphylococcus aureus* in the United States, 2001–2004. J Infect Dis. 2008;197:1226–34. 10.1086/53349418422434

[10] Kaplan SL, Hulten KG, Gonzalez BE, Hammerman WA, Lamberth L, Versalovic J, Three-year surveillance of community-acquired *Staphylococcus aureus* infections in children. Clin Infect Dis. 2005;40:1785–91. 10.1086/43031215909267

[11] Alfaro C, Mascher-Denen M, Fergie J, Purcell K. Prevalence of methicillin-resistant *Staphylococcus aureus* nasal carriage in patients admitted to Driscoll Children’s Hospital. Pediatr Infect Dis J. 2006;25:459–61. 10.1097/01.inf.0000215228.25382.bd16645516

[12] Zaoutis TE, Toltzis P, Chu J, Abrams T, Dul M, Kim J, Clinical and molecular epidemiology of community-acquired methicillin-resistant *Staphylococcus aureus* infections among children with risk factors for health care–associated infection: 2001–2003. Pediatr Infect Dis J. 2006;25:343–8. 10.1097/01.inf.0000207403.67197.cc16567987

[13] Saiman L, O’Keefe M, Graham PL III, Wu F, Said-Salim B, Kreiswirth B, Hospital transmission of community-acquired methicillin-resistant *Staphylococcus aureus* among postpartum women. Clin Infect Dis. 2003;37:1313–9. 10.1086/37902214583864

[14] Popovich KJ, Weinstein RA, Hota B. Are community-associated methicillin-resistant *Staphylococcus aureus* (MRSA) strains replacing traditional nosocomial MRSA strains? Clin Infect Dis. 2008;46:787–94. 10.1086/52871618266611

[15] Seybold U, Kourbatova EV, Johnson JG, Halvosa SJ, Wang YF, King MD, Emergence of community-associated methicillin-resistant *Staphylococcus aureus* USA300 genotype as a major cause of health care–associated blood stream infections. Clin Infect Dis. 2006;42:647–56. 10.1086/49981516447110

[16] Lin YC, Lauderdale TL, Lin HM, Chen PC, Cheng MF, Hsieh KS, An outbreak of methicillin-resistant *Staphylococcus aureus* infection in patients of a pediatric intensive care unit and high carriage rate among health care workers. J Microbiol Immunol Infect. 2007;40:325–34.17712467

[17] Milstone AM, Song X, Beers C, Berkowitz I, Carroll KC, Perl TM. Unrecognized burden of methicillin-resistant *Staphylococcus aureus* and vancomycin-resistant *Enterococcus* carriage in the pediatric intensive care unit. Infect Control Hosp Epidemiol. 2008;29:1174–6. 10.1086/59209318983253

[18] Warren DK, Guth RM, Coopersmith CM, Merz LR, Zack JE, Fraser VJ. Epidemiology of methicillin-resistant *Staphylococcus aureus* colonization in a surgical intensive care unit. Infect Control Hosp Epidemiol. 2006;27:1032–40. 10.1086/50791917006809

[19] Cohen AL, Calfee D, Fridkin SK, Huang SS, Jernigan JA, Lautenbach E, Recommendations for metrics for multidrug-resistant organisms in healthcare settings: SHEA/HICPAC Position paper. Infect Control Hosp Epidemiol. 2008;29:901–13. 10.1086/59174118808340

[20] Horan TC, Andrus M, Dudeck MA. CDC/NHSN surveillance definition of health care-associated infection and criteria for specific types of infections in the acute care setting. Am J Infect Control. 2008;36:309–32. 10.1016/j.ajic.2008.03.00218538699

[21] Farley JE, Stamper PD, Ross T, Cai M, Speser S, Carroll KC. Comparison of the BD GeneOhm methicillin-resistant *Staphylococcus aureus* (MRSA) PCR assay to culture by use of BBL CHROMagar MRSA for detection of MRSA in nasal surveillance cultures from an at-risk community population. J Clin Microbiol. 2008;46:743–6. 10.1128/JCM.02071-0718057129PMC2238090

[22] Murray BE, Singh KV, Heath JD, Sharma BR, Weinstock GM. Comparison of genomic DNAs of different enterococcal isolates using restriction endonucleases with infrequent recognition sites. J Clin Microbiol. 1990;28:2059–63.197776610.1128/jcm.28.9.2059-2063.1990PMC268103

[23] Tenover FC, Arbeit RD, Goering RV, Mickelsen PA, Murray BE, Persing DH, Interpreting chromosomal DNA restriction patterns produced by pulsed-field gel electrophoresis: criteria for bacterial strain typing. J Clin Microbiol. 1995;33:2233–9.749400710.1128/jcm.33.9.2233-2239.1995PMC228385

[24] Lucet JC, Chevret S, Durand-Zaleski I, Chastang C, Regnier B. Prevalence and risk factors for carriage of methicillin-resistant *Staphylococcus aureus* at admission to the intensive care unit: results of a multicenter study. Arch Intern Med. 2003;163:181–8. 10.1001/archinte.163.2.18112546608

[25] Davis KA, Stewart JJ, Crouch HK, Florez CE, Hospenthal DR. Methicillin-resistant *Staphylococcus aureus* (MRSA) nares colonization at hospital admission and its effect on subsequent MRSA infection. Clin Infect Dis. 2004;39:776–82. 10.1086/42299715472807

[26] Jernigan JA, Titus MG, Groschel DH, Getchell-White S, Farr BM. Effectiveness of contact isolation during a hospital outbreak of methicillin-resistant *Staphylococcus aureus.* Am J Epidemiol. 1996;143:496–504.861066510.1093/oxfordjournals.aje.a008770

[27] Khoury J, Jones M, Grim A, Dunne WM Jr, Fraser V. Eradication of methicillin-resistant *Staphylococcus aureus* from a neonatal intensive care unit by active surveillance and aggressive infection control measures. Infect Control Hosp Epidemiol. 2005;26:616–21. 10.1086/50259016092741

[28] Huang SS, Platt R. Risk of methicillin-resistant *Staphylococcus aureus* infection after previous infection or colonization. Clin Infect Dis. 2003;36:281–5. 10.1086/34595512539068

[29] Hidron AI, Kourbatova EV, Halvosa JS, Terrell BJ, McDougal LK, Tenover FC, Risk factors for colonization with methicillin-resistant *Staphylococcus aureus* (MRSA) in patients admitted to an urban hospital: emergence of community-associated MRSA nasal carriage. Clin Infect Dis. 2005;41:159–66. 10.1086/43091015983910

[30] Creech CB II, Kernodle DS, Alsentzer A, Wilson C, Edwards KM. Increasing rates of nasal carriage of methicillin-resistant *Staphylococcus aureus* in healthy children. Pediatr Infect Dis J. 2005;24:617–21. 10.1097/01.inf.0000168746.62226.a415999003

[31] Chen AE, Goldstein M, Carroll K, Song X, Perl TM, Siberry GK. Evolving epidemiology of pediatric *Staphylococcus aureus* cutaneous infections in a Baltimore hospital. Pediatr Emerg Care. 2006;22:717–23. 10.1097/01.pec.0000236832.23947.a017047471

[32] Szczesiul JM, Shermock KM, Murtaza UI, Siberry GK. No decrease in clindamycin susceptibility despite increased use of clindamycin for pediatric community-associated methicillin-resistant *Staphylococcus aureus* skin infections. Pediatr Infect Dis J. 2007;26:852–4. 10.1097/INF.0b013e318124aa5c17721387

[33] Moran GJ, Krishnadasan A, Gorwitz RJ, Fosheim GE, McDougal LK, Carey RB, Methicillin-resistant *S. aureus* infections among patients in the emergency department. N Engl J Med. 2006;355:666–74. 10.1056/NEJMoa05535616914702

[34] Gonzalez BE, Martinez-Aguilar G, Hulten KG, Hammerman WA, Coss-Bu J, Avalos-Mishaan A, Severe staphylococcal sepsis in adolescents in the era of community-acquired methicillin-resistant *Staphylococcus aureus.* Pediatrics. 2005;115:642–8. 10.1542/peds.2004-230015741366

[35] Adem PV, Montgomery CP, Husain AN, Koogler TK, Arangelovich V, Humilier M, *Staphylococcus aureus* sepsis and the Waterhouse-Friderichsen syndrome in children. N Engl J Med. 2005;353:1245–51. 10.1056/NEJMoa04419416177250

[36] Miller LG, Perdreau-Remington F, Bayer AS, Diep B, Tan N, Bharadwa K, Clinical and epidemiologic characteristics cannot distinguish community-associated methicillin-resistant *Staphylococcus aureus* infection from methicillin-susceptible *S. aureus* infection: a prospective investigation. Clin Infect Dis. 2007;44:471–82. 10.1086/51103317243048

[37] Rohr U, Mueller C, Wilhelm M, Muhr G, Gatermann S. Methicillin-resistant *Staphylococcus aureus* whole-body decolonization among hospitalized patients with variable site colonization by using mupirocin in combination with octenidine dihydrochloride. J Hosp Infect. 2003;54:305–9. 10.1016/S0195-6701(03)00140-312919762

[38] Ringberg H, Cathrine Petersson A, Walder M, Hugo Johansson PJ. The throat: an important site for MRSA colonization. Scand J Infect Dis. 2006;38:888–93. 10.1080/0036554060074054617008233

# Research article: Community-associated Methicillin-Resistant *Staphylococcus aureus* Strains in Pediatric Intensive Care Unit^1^

**DOI:** 10.3201/eid1604.090107

**Authors:** Aaron M. Milstone, Karen C. Carroll, Tracy Ross, K. Alexander Shangraw, Trish M. Perl

**Affiliations:** The Johns Hopkins University School of Medicine, Baltimore, Maryland, USA

## Earning CME Credit

To obtain credit, you should first read the journal article. After reading the article, you should be able to answer the following, related, multiple-choice questions. To complete the questions and earn continuing medical education (CME) credit, please go to www.medscapecme.com/journal/eid. Credit cannot be obtained for tests completed on paper, although you may use the worksheet below to keep a record of your answers. You must be a registered user on Medscape.com. If you are not registered on Medscape.com, please click on the New Users: Free Registration link on the left hand side of the website to register. Only one answer is correct for each question. Once you successfully answer all post-test questions you will be able to view and/or print your certificate. For questions regarding the content of this activity, contact the accredited provider, CME@medscape.net. For technical assistance, contact CME@webmd.net. American Medical Association’s Physician’s Recognition Award (AMA PRA) credits are accepted in the US as evidence of participation in CME activities. For further information on this award, please refer to http://www.ama-assn.org/ama/pub/category/2922.html. The AMA has determined that physicians not licensed in the US who participate in this CME activity are eligible for *AMA PRA Category 1 Credits*™. Through agreements that the AMA has made with agencies in some countries, AMA PRA credit is acceptable as evidence of participation in CME activities. If you are not licensed in the US and want to obtain an AMA PRA CME credit, please complete the questions online, print the certificate and present it to your national medical association.

### Article Title: Community-associated Methicillin-Resistant *Staphylococcus aureus* Strains in Pediatric Intensive Care Unit

## CME Questions

**1. Patients in the study cohort found to be colonized with community-acquired methicillin-resistant Staphylococcus aureus (CA-MRSA) were more likely to:**

A. Be adolescent-age boys

B. Have been admitted to a general ward in a hospital in the prior 12 months

C. Be white girls

D. Have been admitted to an intensive care unit in the prior 12 months

**2. Failure to screen a high-risk group, such as children, for MRSA colonization upon admission to a hospital may result in:**

A. Transmission of CA-MRSA strains among hospitalized children

B. Transmission of hospital-acquired (HA)-MRSA strains among children in a community setting

C. Transmission of CA-MRSA strains among children in a community setting

D. Transmission of HA-MRSA strains among hospitalized children

**3. Surveillance cultures done on admission in the study cohort detected:**

A. A large proportion of children colonized with HA-MRSA

B. A small proportion of children infected with CA-MRSA

C. A large proportion of children colonized with CA-MRSA

D. A small proportion of children infected with HA-MRSA

**4. The value of screening patients for MRSA on admission to a hospital is:**

A. Notification of family members to initiate home decolonization regimen

B. Initiation of isolation and contact precautions

C. Early treatment of infection

D. Early initiation of a decolonization regimen

### Activity Evaluation

**Table:** 

1. The activity supported the learning objectives.				
Strongly Disagree				Strongly Agree
1	2	3	4	5
2. The material was organized clearly for learning to occur.				
Strongly Disagree				Strongly Agree
1	2	3	4	5
3. The content learned from this activity will impact my practice.				
Strongly Disagree				Strongly Agree
1	2	3	4	5
4. The activity was presented objectively and free of commercial bias.				
Strongly Disagree				Strongly Agree
1	2	3	4	5
